# Caregiver burden and psychological distress among family caregivers of people with dementia: the mediating roles of family resilience and sense of coherence

**DOI:** 10.3389/fpubh.2026.1999212

**Published:** 2026-10-30

**Authors:** Shuang Zhang, Yanming Ma, Liniu Pan, Mengli Yang

**Affiliations:** 1 Department of Neurology, Henan Provincial People’s Hospital, Henan Provincial Key Medicine Laboratory of Nursing, Zhengzhou University People’s Hospital, Zhengzhou, China; 2 School of English Studies, Tianjin Foreign Studies University, Tianjin, China; 3 School of Nursing, Zhengzhou Health Vocational College, Zhengzhou, China

**Keywords:** caregiver burden, dementia, family resilience, psychological distress, sense of coherence

## Abstract

**Background:**

Family caregivers of people with dementia often experience substantial psychological distress due to the burden associated with long-term caregiving. Although previous studies have identified an association between caregiver burden and psychological distress, the roles of adaptive resources in this association remain insufficiently understood. This study aimed to examine the association between caregiver burden and psychological distress and to explore the independent and sequential mediating roles of family resilience and sense of coherence (SOC) among family caregivers of people with dementia.

**Methods:**

In this cross-sectional study, we recruited 305 family caregivers of people with dementia from two tertiary hospitals in Henan Province, China. Data were collected using the Chinese versions of the Zarit Burden Interview, Family Resilience Assessment Scale, Sense of Coherence Scale, and 10-item Kessler Psychological Distress Scale. Pearson’s correlation analysis was conducted to examine the associations among caregiver burden, family resilience, SOC, and psychological distress. The direct and indirect effects were examined using sequential mediation analysis with Model 6 of the SPSS PROCESS macro version 4.2.

**Results:**

Significant correlations were found between the measured variables (each *p* < 0.001). Family resilience and SOC each demonstrated significant indirect associations in the relationship between caregiver burden and psychological distress. In addition, a significant sequential indirect association through family resilience and SOC was identified, accounting for 5.4% of the total effect.

**Conclusion:**

Family resilience and SOC were important mediating resources in the association between caregiver burden and psychological distress among family caregivers of people with dementia. The findings highlight the interconnected roles of family-level and individual-level psychological resources in caregivers’ psychological adaptation to dementia caregiving stress. Interventions targeting family resilience and SOC may represent potential approaches for supporting caregivers’ psychological well-being, warranting further investigation through longitudinal and intervention studies.

## Introduction

As populations age worldwide, dementia has become an increasingly important public health concern (1). The number of people living with dementia globally is projected to increase from 57.4 million in 2019 to 152.8 million by 2050 (2). China bears a substantial share of the global dementia burden, accounting for nearly one-third of all cases worldwide (3). In 2021, 16.99 million people were living with dementia in China, creating considerable demands for long-term care and support (3). Owing to the progressive and irreversible nature of the disease, people with dementia often require extensive assistance with daily activities and health management (4, 5). Under the influence of traditional filial piety and family-centered culture, the majority of people with dementia are cared for at home by family members (6). Consequently, family caregivers assume primary responsibility for providing long-term care and support throughout the disease trajectory, making their health and well-being an important public health concern.

Caregiver burden is one of the most prevalent and significant challenges faced by family caregivers of people with dementia. It refers to the physical, emotional, social, and financial stressors experienced as a result of caregiving responsibilities (7). Growing evidence suggests that prolonged caregiving burden not only compromises caregivers’ physical health but also places them at increased risk of adverse psychological outcomes (8, 9). Among these, psychological distress, characterized by symptoms of anxiety, depression, and emotional suffering, is one of the most common mental health problems experienced by dementia caregivers (10, 11). High levels of psychological distress among caregivers is associated with poorer quality of life, reduced caregiving capacity, and adverse care outcomes (12, 13). A substantial body of research has demonstrated a positive association between caregiver burden and psychological distress (14–16). Although previous studies have explored potential explanatory factors for psychological distress among dementia caregivers, the roles of psychosocial resources in the association between caregiver burden and psychological distress remain insufficiently understood.

In this context, exploring potential protective factors is essential to further elucidate this relationship. Family resilience has been increasingly recognized as a key protective factor in the context of caregiving stress, defined as the dynamic capacity of families to utilize internal strengths and external resources to withstand and recover from significant stress or chronic adversity while maintaining or restoring family functioning (17). Previous research has highlighted the importance of family-level factors in dementia caregiving, suggesting that the family functions as a key system shaping caregivers’ adaptation to chronic stress and psychological outcomes (18). During long-term caregiving, caregiver burden in families of people with dementia may be associated with declines in family adaptation and functional maintenance. In contrast, families with high resilience may maintain stable functioning even under stress, which may be associated with variations in caregivers’ psychological responses to caregiver burden (19). Previous studies have shown that higher levels of family resilience are associated with better family resource integration and lower levels of caregiver psychological distress (20, 21). In this regard, family resilience, as a family-level protective resource, may help explain differences in caregivers’ psychological responses to caregiving burden. Therefore, family resilience may represent an important psychological resource associated with the relationship between caregiver burden and psychological distress.

In addition to family-level resources, individual psychological resources also play a critical role in shaping caregivers’ responses to stress. Sense of coherence (SOC), derived from Antonovsky’s salutogenic theory, reflects an individual’s capacity to perceive life as comprehensible, manageable, and meaningful (22, 23). In the context of dementia caregiving, SOC has been identified as an important personal psychological resource that may help caregivers better understand and cope with caregiving demands (24). Previous studies have shown that higher caregiver burden is linked to lower levels of SOC among caregivers (25–27). Moreover, SOC has been shown to be associated with weaker adverse psychological outcomes of caregiver strain and with better psychological adaptation to long-term caregiving (28). Furthermore, higher levels of SOC are related to lower psychological distress and better health-related quality of life (29–31). Taken together, SOC may represent an important psychological resource linking caregiver burden to psychological distress and serve as a potential mediator in this relationship.

Recent evidence has suggested that family resilience and SOC are closely related in dementia caregiving (25). Previous research has also indicated that family resilience plays a mediating role in the association between caregiver burden and SOC, highlighting its importance in maintaining caregivers’ psychological resources under caregiving stress (25). However, although previous studies have examined multiple mechanisms linking caregiver burden and psychological distress, including coping resources, social support, self-efficacy, and family functioning, important gaps remain (14, 15). Specifically, the sequential interplay between family-level and individual-level psychological resources has rarely been examined within a unified framework for explaining caregivers’ psychological distress. Examining these two resources simultaneously may provide a more comprehensive understanding of how family- and individual-level psychological resources jointly shape caregivers’ psychological adaptation to caregiving burden. Therefore, the present study aims to investigate the associations among caregiver burden, family resilience, SOC, and psychological distress, and to examine the potential chain mediating effect of family resilience and SOC, thereby providing preliminary evidence to inform future intervention research.

## Conceptual framework

This study is grounded in the Stress Process Model (SPM), which conceptualizes caregiving as a dynamic stress process in which caregiving stressors influence psychological outcomes through psychosocial resources (32). Within this framework, caregiver burden represents a primary chronic stressor that is associated with adverse psychological outcomes, including psychological distress. However, the impact of caregiving stress is influenced by protective resources operating at both the family and individual levels. Family resilience, as a family-level resource, reflects the capacity of families to maintain functioning and adapt to caregiving challenges, and may represent a resource associated with caregivers’ adaptation to caregiving challenges (17). At the individual level, SOC represents an intrapersonal psychological resource that shapes caregivers’ cognitive appraisal of caregiving demands and facilitates psychological adaptation (22, 23). Building on the SPM, family-level and individual-level resources may jointly shape caregivers’ psychological adaptation to caregiving stress. In this regard, family resilience may be associated with higher levels of SOC by providing a supportive and well-functioning family environment, suggesting a potential sequential indirect association between caregiver burden and psychological distress. The conceptual framework of this study is presented in Figure 1.

**Figure 1:**
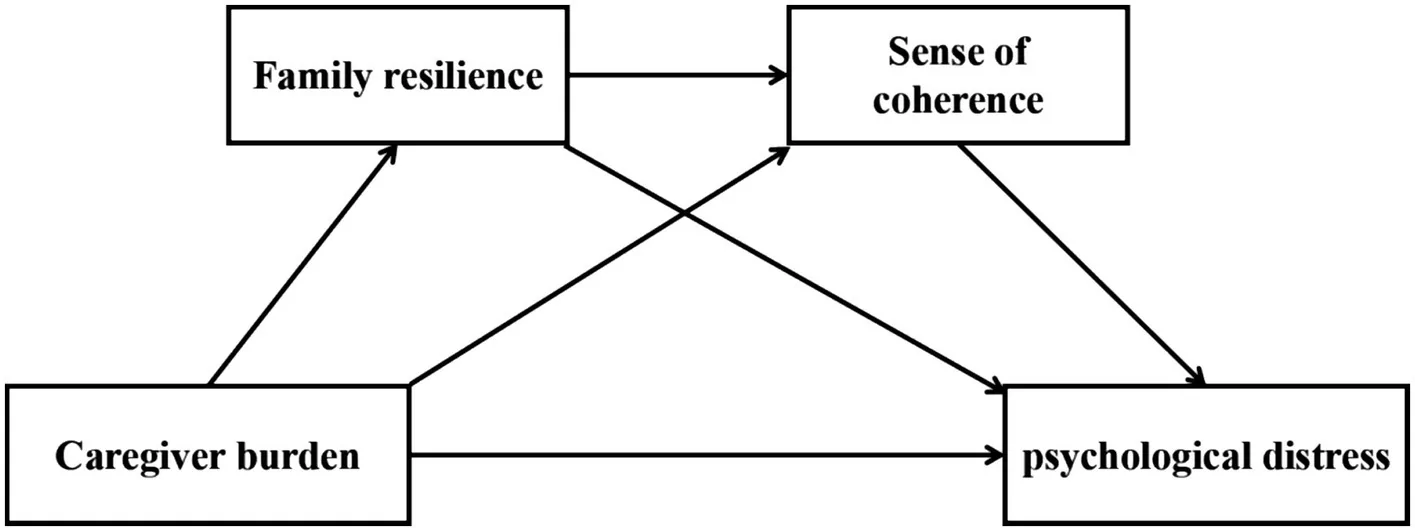
Hypothesized conceptual model of the chain mediation.

Based on the conceptual framework, we proposed the following hypotheses:

*H1*: Caregiver burden is positively associated with psychological distress among family caregivers of people with dementia.

*H2*: Family resilience mediates the relationship between caregiver burden and psychological distress among family caregivers of people with dementia.

*H3*: SOC mediates the association between caregiver burden and psychological distress among family caregivers of people with dementia.

*H4*: Family resilience and SOC sequentially mediate the association between caregiver burden and psychological distress among family caregivers of people with dementia.

## Methods

### Study design

From April to October 2025, a cross-sectional study using convenience sampling was conducted among family caregivers of people with dementia in two tertiary hospitals in Henan Province, China. The study was reported in accordance with the Strengthening the Reporting of Observational Studies in Epidemiology (STROBE) guidelines (33).

### Participants

Informal caregivers of people with dementia were eligible to participate if they: (1) were aged 18 years or older; (2) were the primary family caregiver of a person diagnosed with dementia by a neurologist or psychiatrist according to established diagnostic criteria; (3) had provided unpaid care for at least 3 months; and (4) were able to communicate in Chinese and were willing to participate in the study by providing written informed consent. Caregivers were excluded if they: (1) had severe cognitive impairment; (2) had severe physical or mental disorders; (3) had severe visual or hearing impairments that could interfere with questionnaire completion.

The required sample size was estimated using G*Power version 3.1 based on a multiple linear regression model (34). Assuming a medium effect size (*f*^2^ = 0.15), a significance level of 0.05, a statistical power of 0.95, and 18 predictors, the minimum required sample size was 213 participants. Considering a potential 20% dropout rate due to incomplete questionnaires or non-response, at least 267 participants were required. A total of 305 participants were included in the final analysis, which exceeded the minimum sample size requirement.

### Data collection

Prior to data collection, all investigators received standardized training to ensure consistency in participant recruitment and questionnaire administration. Potential participants were identified through medical records and referrals from neurologists and nursing staff in the neurology departments of two tertiary hospitals. Before participation, the researchers explained the study objectives, procedures, and voluntary nature of participation, and written informed consent was obtained from all participants. Questionnaires were completed independently in a quiet environment. For caregivers with limited literacy or reading difficulties, trained investigators provided standardized assistance by reading the questionnaire items aloud without interpretation or leading responses. Caregiver demographic information was collected via self-report, whereas demographic and clinical information of people with dementia was obtained from medical records and caregiver reports. All questionnaires were checked immediately after completion for completeness and logical consistency, and missing items were clarified whenever possible. A total of 305 valid questionnaires were included in the final analysis.

### Measures

#### Sociodemographic and clinical characteristics

Participants’ sociodemographic and clinical information was collected using a structured questionnaire and medical record review. Caregiver characteristics included age, sex, marital status, educational level, employment status, relationship to the person with dementia, co-residence status, duration of caregiving, and average daily caregiving hours. Information on people with dementia included age, gender, dementia type, disease duration, dementia severity, and comorbidities. Dementia severity was assessed using the Clinical Dementia Rating (CDR) scale, and CDR scores were obtained from medical records.

#### Zarit burden interview

Caregiver burden was assessed using the Chinese version of 22-item Zarit Burden Interview (ZBI) scale (35). The scale consists of two dimensions: personal burden and responsibility burden. Each item is rated on a five-point Likert scale ranging from 0 (never) to 4 (nearly always), yielding a total score ranging from 0 to 88, with higher scores indicating greater caregiver burden. The reliability and validity of the instrument have been supported in previous studies (36, 37). In the present study, the Cronbach’s alpha of the scale was 0.938.

#### 10-item Kessler psychological distress scale

Psychological distress was measured using the Chinese version of the 10-item Kessler Psychological Distress Scale (K10) (38). The K10 is a widely used self-report instrument designed to assess non-specific psychological distress, including symptoms of anxiety and depression experienced during the previous 4 weeks. Each item is rated on a five-point Likert scale ranging from 1 (none of the time) to 5 (all of the time). Total scores range from 10 to 50, with higher scores reflecting more severe psychological distress. The Chinese version of the K10 has demonstrated good reliability and validity in measuring the psychological distress in the Chinese population (39). The Cronbach’s alpha of the scale was 0.944 in the current study.

#### Family resilience assessment scale-Chinese version

Family resilience among caregivers was assessed using the Chinese version of the Family Resilience Assessment Scale (FRAS-C) (40). The FRAS-C consists of 32 items covering three dimensions: Family Communication and Problem Solving (FCPS), Maintaining a Positive Outlook (MPO), and Utilizing Social Resources (USR). Responses are scored on a four-point Likert scale ranging from 1 (strongly disagree) to 4 (strongly agree), with total scores spanning 32 to 128. Higher total scores indicate greater family resilience. The FRAS-C has demonstrated satisfactory psychometric properties among Chinese family caregivers (41). In our study, the Cronbach’s alpha of this scale was 0.969.

#### Sense of coherence scale

We used the Chinese version of the 13-item Sense of Coherence Scale (SOC-13) to measure overall sense of coherence of caregivers (42). The SOC-13 comprises three dimensions: comprehensibility, manageability, and meaningfulness. It is rated on a seven-point Likert scale, and five items are negatively worded and reverse-coded before scoring. Total scores are calculated by summing all item responses, yielding a score ranging from 13 to 91, with higher scores indicating a stronger sense of coherence. The Chinese version of the SOC-13 demonstrated adequate internal consistency, with a Cronbach’s alpha of 0.939 (25). The Cronbach’s alpha of the scale was 0.846 in our study.

#### Statistical analysis

Statistical analysis for this research was conducted using IBM SPSS Statistics version 26.0 and SPSS PROCESS macro version 4.2 (Model 6). Descriptive statistics, including means, standard deviations (SDs), frequencies, and percentages, were used to describe the sample characteristics and main study variables. Independent-sample t-tests and analysis of variance were used to analyze the effects of different demographic characteristics on psychological distress. Pearson’s correlation analysis was conducted to assess the associations between caregiver burden, family resilience, SOC, and psychological distress. Multicollinearity among predictors was assessed using variance inflation factors (VIFs) and tolerance values. Common method bias was examined using Harman’s single-factor test. The chain mediation model was examined using Model 6 of the SPSS PROCESS macro Version 4.2. In the mediation model, caregiver burden was specified as the independent variable, psychological distress as the dependent variable, and family resilience and sense of coherence as sequential mediators. Covariates included dementia type, dementia severity, number of comorbidities, caregiver age, and caregiving duration. These variables were initially identified based on their statistically significant associations with psychological distress in univariate analyses, and their relevance was considered in light of the conceptual framework and established literature. The mediation effects were tested using the bias-corrected percentile Bootstrap method with 5,000 resamples. When the 95% confidence interval did not include zero, the effect was deemed statistically significant. All statistical tests were two-tailed, with statistical significance set at *p* < 0.05.

#### Ethical considerations

This study adhered to the ethical principles of the Declaration of Helsinki and was approved by the Medical Ethics Committee of Henan Provincial People’s Hospital (Approval No: 2024–217). Written informed consent was obtained from all participants included in the study. All data is anonymous and strictly confidential.

## Results

### Sample characteristics

A total of 305 family caregivers of people with dementia were included in the final analysis. Table 1 summarizes the characteristics of the participants and their relationships with psychological distress. The mean age of caregivers was 52.71 years (SD = 12.70), with 118 males (38.7%) and 187 females (61.3%). Most caregivers were married (96.1%), lived with the person with dementia (80.3%), and were adult children of the patients (54.8%). Over half had cared for patients for one to 3 years (56.7%), and 41.0% reported providing care for more than 12 h per day. Among people with dementia, the mean age was 70.78 years (SD = 8.11), and more than half were male (51.8%). The majority of patients were diagnosed with Alzheimer’s disease (39.3%), followed by vascular dementia (31.2%) and other dementia subtypes (29.5%). The severity of the disease was mainly moderate in 195 (63.9%) cases. Univariate analyses showed that type of dementia, disease severity, number of comorbidities, caregiver age, and duration of caregiving had significant differences in the caregiver psychological distress (*P* < 0.05). Detailed results are presented in Table 1.

**Table 1:** Sociodemographic and clinical characteristics of study participants (*N* = 305).

Variables	*N* (%)	Psychological distress (Mean ± SD)	t/F	*p*
Patient characteristics				
Age (years)			1.038	0.355
60 ~ 69	133 (43.6)	21.32 ± 7.52		
70 ~ 79	120 (39.4)	21.43 ± 7.37		
≥ 80	52 (17.0)	23.02 ± 7.84		
Gender			−1.184	0.237
Male	158 (51.8)	21.16 ± 7.40		
Female	147 (48.2)	22.18 ± 7.63		
Type of dementia			6.165	< 0.001^***^
Alzheimer’s disease	120 (39.3)	22.13 ± 7.18		
Vascular dementia	95 (31.2)	21.35 ± 7.82		
Mixed dementia	52 (17.0)	18.58 ± 6.97		
Other	38 (12.5)	25.16 ± 7.03		
Disease severity			7.755	0.001^**^
Mild	73 (23.9)	18.74 ± 7.01		
Moderate	195 (63.9)	22.44 ± 7.53		
Severe	37 (12.2)	23.30 ± 7.07		
Duration of illness (years)			1.667	0.191
< 1	46 (15.1)	20.46 ± 7.89		
1 ~ 3	163 (53.4)	22.37 ± 7.11		
> 3	96 (31.5)	21.02 ± 7.95		
Number of comorbidities			−3.796	< 0.001^***^
< 3	235 (77.0)	20.78 ± 7.56		
≥ 3	70 (23.0)	24.59 ± 6.61		
Caregiver characteristics				
Age (years)			2.707	0.007^**^
< 60	180 (59.0)	22.62 ± 7.38		
≥60	125 (41.0)	20.27 ± 7.53		
Gender			0.103	0.918
Male	118 (38.7)	21.71 ± 7.35		
Female	187 (61.3)	21.62 ± 7.64		
Marital status			−0.240	0.811
Unmarried	12 (3.9)	22.17 ± 8.17		
Married	293 (96.1)	21.63 ± 7.50		
Education level			0.961	0.411
Primary school or below	55 (18.0)	20.73 ± 7.64		
Middle school	95 (31.2)	22.35 ± 7.14		
High school	66 (21.6)	20.79 ± 7.00		
College or above	89 (29.2)	22.13 ± 8.18		
Employment status			1.148	0.252
Unemployed or retired	156 (51.1)	21.17 ± 7.31		
Employed	149 (48.9)	22.16 ± 7.72		
Relationship with patients			0.429	0.651
Spouse	128 (42.0)	21.32 ± 7.44		
Children	167 (54.8)	21.81 ± 7.72		
Other family members	10 (3.2)	23.40 ± 4.81		
Living with patients			0.619	0.536
Yes	245 (80.3)	21.79 ± 7.47		
No	60 (19.7)	21.12 ± 7.73		
Duration of caregiving (years)			3.740	0.025^*^
<1	52 (17.0)	19.77 ± 8.36		
1 ~ 3	173 (56.7)	21.45 ± 6.93		
>3	80 (26.3)	23.33 ± 7.91		
Care time per day (hours)			0.350	0.705
<8	107 (35.1)	21.38 ± 6.61		
8 ~ 12	73 (23.9)	21.32 ± 8.75		
>12	125 (41.0)	22.09 ± 7.52		

*p < 0.05; **p < 0.01; ***p < 0.001.

### Common method bias and multicollinearity

Harman’s single-factor test was conducted to assess common method bias. The results showed that there were 14 factors with characteristic roots greater than 1, and the first factor accounted for 32.2% of the variance, less than the critical standard of 40% (43). This suggests that common method bias was not a significant issue in the present study.

Multicollinearity diagnostics for the regression model showed that VIF values ranged from 1.024 to 2.453, and tolerance values ranged from 0.408 to 0.976, indicating no substantial multicollinearity among the predictors.

### Descriptive statistics and correlations of caregiver burden, family resilience, SOC, and psychological distress

Table 2 presents the descriptive statistics and Pearson’s correlation coefficients for the study variables. All associations were statistically significant (*p* < 0.001). The results showed that caregiver burden was negatively correlated with family resilience (*r* = −0.599, *p* < 0.001) and SOC (*r* = −0.719, *p* < 0.001), while it was positively correlated with psychological distress (*r* = 0.782, *p* < 0.001). Family resilience was positively associated with SOC (*r* = 0.525, *p* < 0.001) and negatively associated with psychological distress (*r* = −0.581, *p* < 0.001). In addition, SOC was negatively correlated with psychological distress (*r* = −0.836, *p* < 0.001).

**Table 2:** Descriptive statistics and correlations of all variables (*N* = 305).

Variable	Mean	SD	Caregiver burden	Family resilience	SOC	Psychological distress
Caregiver burden	32.50	18.25	1			
Family resilience	99.39	14.42	−0.599^***^	1		
SOC	57.29	10.31	−0.719^***^	0.525^***^	1	
Psychological distress	21.66	7.52	0.782^***^	−0.581^***^	−0.836^***^	1

***p < 0.001. SOC, Sense of Coherence.

### Chain mediation effects analysis

The chain mediation model for family resilience and SOC is shown in Figure 2. Variables showing significant associations with psychological distress in the univariate analyses were entered as covariates in the mediation model, including type of dementia, disease severity, number of comorbidities, caregiver age, and duration of caregiving. As shown in Table 3, caregiver burden was negatively associated with family resilience (*β* = −0.557, *p* < 0.001). Both caregiver burden (*β* = −0.629, *p* < 0.001) and family resilience (*β* = 0.137, *p* < 0.01) were significantly related to SOC. In addition, caregiver burden (*β* = 0.333, *p* < 0.001), family resilience (*β* = −0.089, *p* < 0.05) and SOC (*β* = −0.545, *p* < 0.001) were each significantly associated with psychological distress. These findings indicate that caregiver burden is associated with psychological distress both directly and indirectly through family resilience and SOC.

**Figure 2:**
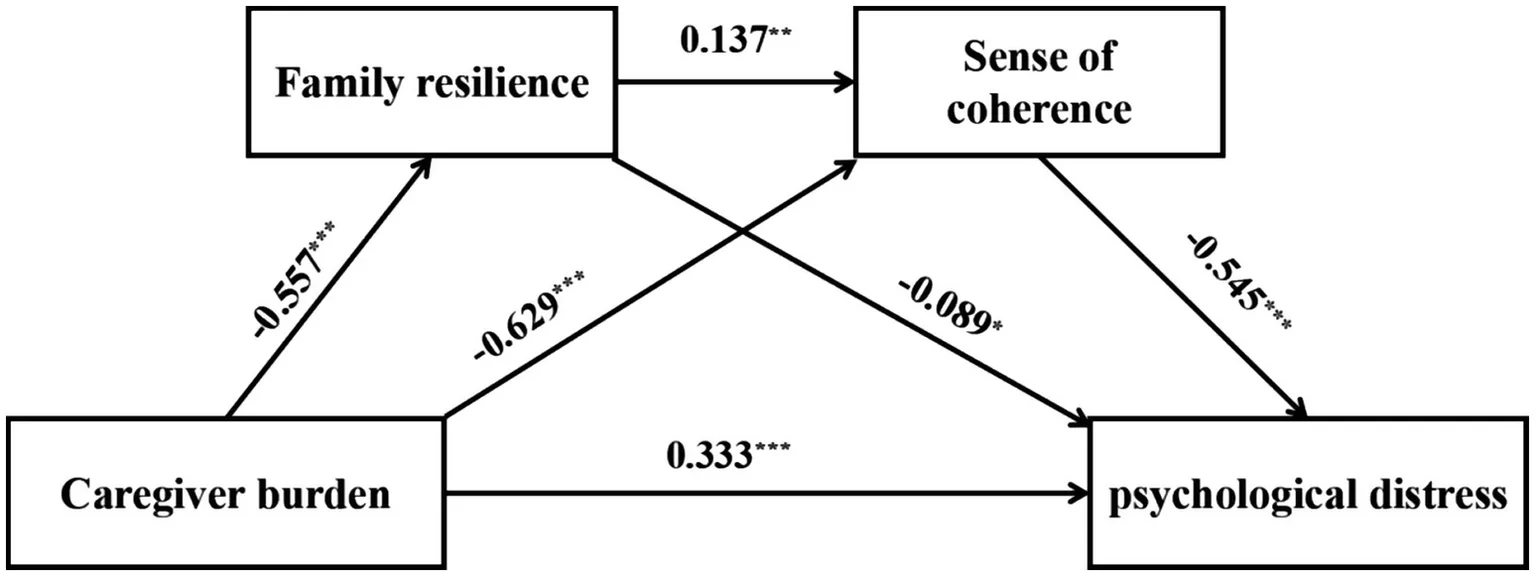
The chain mediation model. Path coefficients were shown in the standardized regression coefficient.* *p* < 0.05, ** *p* < 0.01, *** *p* < 0.001.

**Table 3:** Regression results of the chain mediation model.

Variables	Family resilience				SOC			Psychological distress				
	B	*β*	SE	*t*	B	*β*	SE	*t*	B	*β*	SE	*t*
Type of dementia	−0.439	−0.031	0.644	−0.682	−0.295	−0.030	0.400	−0.738	0.177	0.024	0.202	0.878
Disease severity	−2.224	−0.037	1.165	−1.908	−0.965	−0.055	0.727	−1.328	0.388	0.031	0.368	1.055
Number of comorbidities	−1.343	−0.039	1.714	−0.784	0.257	0.011	1.064	0.242	0.282	0.016	0.537	0.526
Duration of caregiving	−1.414	−0.064	1.108	−1.276	−0.328	−0.021	0.689	−0.477	−0.544	−0.047	0.348	−1.563
Caregiver age	1.132	0.039	1.357	0.834	−0.251	−0.012	0.843	−0.298	−0.771	−0.051	0.425	−1.813
Caregiver burden	−0.441	−0.557	0.038	−11.631^***^	−0.355	−0.629	0.028	−12.534^***^	0.137	0.333	0.018	7.757^***^
Family resilience					0.098	0.137	0.036	2.718^**^	−0.046	−0.089	0.018	−2.528^*^
SOC									−0.398	−0.545	0.029	−13.571^***^
*R* ^2^	0.380				0.535				0.778			
F	30.431^***^				48.743^***^				129.586^***^			

*p < 0.05; **p < 0.01; ***p < 0.001. SOC, Sense of Coherence; B, unstandardized coefficients; β, standardized coefficients; SE, Standard error.

As presented in Table 4, the total effect [Effect = 0.316, 95% CI (0.286, 0.346)], direct effect [Effect = 0.137, 95% CI (0.102, 0.172)], and total indirect effect [Effect = 0.179, 95% CI (0.149, 0.208)] of caregiver burden on psychological distress were all statistically significant. Further decomposition of the total indirect effect revealed three significant specific indirect pathways: via family resilience alone [Effect = 0.020, 95% CI (0.003, 0.038)], accounting for 6.3% of the total effect; via SOC alone [Effect = 0.141, 95% CI (0.115, 0.168)], accounting for 44.6% of the total effect; and via the sequential pathway from family resilience to SOC [Effect = 0.017, 95% CI (0.004, 0.032)], accounting for 5.4% of the total effect. The three indirect pathways collectively accounted for 56.6% of the total effect.

**Table 4:** Total, direct and indirect effects of caregiver burden on psychological distress.

Model pathways	Effect value	BootSE	BootLLCI	BootULCI
Total effect	0.316	0.015	0.286	0.346
Direct effect	0.137	0.018	0.102	0.172
Total indirect effect	0.179	0.015	0.149	0.208
Indrect 1: Caregiver burden → Family resilience → Psychological distress	0.020	0.009	0.003	0.038
Indrect 2: Caregiver burden → Sense of coherence → Psychological distress	0.141	0.014	0.115	0.168
Indrect 3: Caregiver burden → Family resilience → Sense of coherence → Psychological distress	0.017	0.007	0.004	0.032

Effect values were unstandardized coefficients based on 5,000 bootstrap samples. BootSE, bootstrap standard error; BootLLCI, Lower limit of 95% bootstrap confidence interval; BootULCI, Upper limit of 95% bootstrap confidence interval.

## Discussion

This study examined the sequential mediating roles of family resilience and SOC in the association between caregiver burden and psychological distress among family caregivers of people with dementia. The findings showed that caregiver burden was significantly associated with psychological distress both directly and indirectly through family resilience and SOC. In addition to their independent mediating roles, family resilience and SOC also formed a significant sequential indirect association. Among the three specific indirect effects, the mediating effect of SOC accounted for the largest proportion of the total effect. These findings extend current understanding of the psychosocial resources involved in the association between caregiver burden and psychological distress and may provide a basis for future intervention research to support the psychological well-being of family caregivers of people with dementia.

Consistent with prior studies (14–16), the present study established a significant positive association between caregiver burden and psychological distress among family caregivers of people with dementia. This finding may reflect the cumulative psychological strain experienced during prolonged caregiving, which is often accompanied by sustained emotional strain and increased psychological vulnerability over time (44, 45). Notably, more than half of the total statistical effect was accounted for by the estimated indirect effects, suggesting that the association between caregiver burden and psychological distress may also be related to caregivers’ adaptive resources. This interpretation is consistent with the SPM, which proposes that the impact of chronic caregiving stress depends on adaptive resources rather than stress exposure alone (32). Within the Chinese cultural context, where caring for older family members is widely regarded as a familial responsibility rooted in filial piety, prolonged caregiving demands may further intensify caregivers’ psychological strain when adequate supportive resources are lacking (6). Therefore, interventions should not only aim to alleviate caregiver burden but also strengthen caregivers’ adaptive resources to promote psychological well-being.

The present study identified a statistically significant indirect association involving family resilience in the relationship between caregiver burden and psychological distress, supporting Hypothesis 2. This finding suggests that family resilience may help account for part of the observed association between caregiver burden and psychological distress. Caregiver burden may represent a sustained family-level stressor that challenges the family’s ability to adapt to the ongoing demands of dementia care (32, 46). When the family’s adaptive capacity becomes insufficient, caregiving stress may be less effectively managed at the family level and more likely to be experienced at the individual level, alongside increased perceived responsibility and psychological distress (18, 46). This interpretation extends previous evidence by suggesting that family resilience functions not merely as a protective family characteristic, but as a dynamic adaptive process related to differences in caregivers’ psychological outcomes. Although the mediating effect of family resilience was statistically significant, its magnitude was relatively modest, indicating that its role in linking caregiver burden and psychological distress may primarily reflect a contextual level of family adaptation within the caregiving system.

The present study further demonstrated the mediating role of SOC in the association between caregiver burden and psychological distress, suggesting that this association may be partly explained by caregivers’ SOC. According to Antonovsky’s salutogenic theory, individuals are continuously exposed to stressors and rely on available resources to maintain psychological equilibrium (22, 23). When perceived caregiving demands exceed available coping resources, the balance between stressors and resources may become disrupted, which is reflected in lower SOC levels (47). In dementia caregiving, persistent uncertainty, progressive functional decline, and fluctuating care demands may undermine caregivers’ ability to maintain a coherent understanding of caregiving experiences (48, 49), which may in turn be associated with greater psychological distress. This interpretation is consistent with previous evidence showing that SOC mediates the association between caregiving strain and adverse psychological outcomes among family caregivers (28), while further extending this evidence to family caregivers of people with dementia. Notably, the indirect effect involving SOC was larger than that involving family resilience. This difference may reflect the distinct roles of individual-level and family-level resources in their associations with psychological distress. While family resilience reflects the broader adaptive capacity of the caregiving system, SOC is more closely linked to how caregiving stressors are perceived and cognitively organized at the individual level, which may account for its larger indirect effect. However, the strong negative correlation between SOC and psychological distress warrants cautious interpretation. Although multicollinearity diagnostics did not indicate substantial multicollinearity among the predictors, the strong association may partly reflect conceptual overlap between the two constructs. SOC is conceptualized as a general orientation toward life characterized by comprehensibility, manageability, and meaningfulness, whereas psychological distress reflects negative emotional symptoms. Thus, although the two constructs are conceptually distinct, they may be closely related. The observed association should therefore be interpreted with caution. Future studies using longitudinal designs and complementary measurement approaches are needed to further clarify the relationship between these constructs.

The present study also identified a statistically significant sequential indirect association involving family resilience and SOC in the relationship between caregiver burden and psychological distress, supporting Hypothesis 4. This finding suggests that family-level and individual-level adaptive resources may be interconnected in the context of dementia caregiving. Consistent with the Stress Process Model, caregivers’ psychological adaptation to chronic stress may be understood through the interplay between stressors and available resources (32). Within this framework, family resilience and SOC may represent resources operating at different levels of the caregiving system (17, 23). The proposed ordering of family resilience before SOC was based on the theoretical distinction between family-level adaptive capacity and individual-level appraisal of stressful experiences. Specifically, family resilience reflects a family’s capacity to maintain or restore functioning and coordinate available resources, whereas SOC reflects an individual’s perceptions of the comprehensibility, manageability, and meaningfulness of life experiences. A supportive and adaptive family environment may therefore be associated with how caregivers perceive and make sense of caregiving-related demands (25, 50). This conceptual relationship provides a theoretical rationale for examining a sequential indirect association from family resilience to SOC. However, because all variables were measured concurrently, the observed statistical indirect association does not establish that family resilience precedes SOC temporally or causally. Alternative directions of association, including the possibility that SOC is associated with caregivers’ perceptions of family resilience, cannot be excluded. Accordingly, the sequential indirect effect should be interpreted as a pattern of association consistent with the proposed theoretical model rather than evidence of a confirmed temporal sequence. Overall, these findings highlight the interconnected roles of family- and individual-level resources in understanding caregivers’ psychological responses to dementia caregiving stress.

### Implication for practice

This study provides potential implications for clinical assessment and future intervention research concerning the psychological well-being of family caregivers of people with dementia. Our findings suggest that family resilience and SOC represent important family-level and individual-level psychological resources associated with caregivers’ psychological distress, highlighting the need for caregiver assessment beyond caregiver burden alone. Routine clinical practice may incorporate assessments of these adaptive resources to identify caregivers who are more vulnerable to psychological distress. Furthermore, intervention programs could consider a multilevel approach that simultaneously addresses family and individual psychological resources. At the family level, resilience-enhancing interventions, including psychoeducation, mindfulness-based approaches, and CBT-based interventions, may help strengthen adaptive capacities within the caregiving system and provide a potential pathway for supporting family resilience (51). At the individual level, SOC-oriented interventions may incorporate salutogenic approaches, cognitive-behavioral strategies, and self-management techniques to support caregivers’ adaptation to caregiving challenges (52). Given that the indirect association involving SOC was numerically larger than that involving family resilience in the present model, this finding should not be interpreted as evidence that SOC-focused interventions would be more effective. Future intervention studies could examine whether approaches targeting caregivers’ SOC are associated with improvements in psychological well-being. Integrating family-based and individual-focused strategies may provide a promising approach for future intervention research aimed at supporting caregivers’ long-term psychological well-being. Moreover, interventions should be culturally tailored to the Chinese caregiving context by leveraging family values such as filial piety and family harmony to strengthen supportive family environments while simultaneously fostering caregivers’ individual psychological resources. The effectiveness of these approaches in reducing psychological distress among family caregivers of people with dementia requires further evaluation.

### Limitations

The limitations of this study are as follows. First, the cross-sectional design limits our ability to infer causal relationships among caregiver burden, family resilience, SOC, and psychological distress. Future longitudinal studies are needed to further clarify the temporal and causal relationships among these variables. Second, convenience sampling from two tertiary hospitals may limit the generalizability of the findings. Future studies should include samples from multiple geographic regions and diverse levels of hospitals to improve the representativeness of the findings. Third, this study focused on family resilience and SOC as key adaptive resources within the caregiving process; however, other contextual and individual factors, such as social support, financial strain, coping styles, and caregiver personality characteristics, may also be associated with caregivers’ psychological adaptation. Future research incorporating a broader range of stressors and resources may provide a more comprehensive understanding of the factors associated with psychological distress among dementia caregivers. Fourth, although family resilience was conceptualized as a family-level resource, it was assessed from the caregiver’s perspective. Therefore, the findings may not fully capture the complexity of family resilience as a systemic process involving multiple family members. Future studies using dyadic or multi-informant designs, including perspectives from care recipients and other family members, may further clarify the role of family-level resources in caregiver adaptation. Fifth, all variables were self-reported, and the high correlation between SOC and psychological distress (r = −0.836) may partly reflect common method bias and construct overlap. Therefore, these associations should be interpreted with caution. Future research using multi-source data, objective indicators, or longitudinal designs is warranted.

## Conclusion

This study provides further insights into the relationships among caregiver burden, family resilience, SOC, and psychological distress among family caregivers of people with dementia. The findings suggest that family resilience and SOC are important adaptive resources associated with caregivers’ psychological distress, with the indirect association involving SOC being numerically larger in the tested model. Moreover, the sequential mediation analysis identified an indirect association involving both family resilience and SOC, highlighting the potential relevance of psychological resources at both the family and individual levels in understanding caregivers’ psychological distress. Given the cross-sectional design, these findings should be interpreted as statistical associations rather than evidence of causal relationships or temporal ordering. Future longitudinal research is needed to further clarify the relationships among these variables and examine how psychological resources at both levels relate to caregivers’ psychological well-being over time.

## Data Availability

The raw data supporting the conclusions of this article will be made available by the authors, without undue reservation.
